# Estimating the impact of nutrition and physical activity policies with quasi-experimental methods and simulation modelling: an integrative review of methods, challenges and synergies

**DOI:** 10.1093/eurpub/ckac051

**Published:** 2022-11-29

**Authors:** Karl M F Emmert-Fees, Sara Capacci, Franco Sassi, Mario Mazzocchi, Michael Laxy

**Affiliations:** Institute of Health Economics and Health Care Management, Helmholtz Zentrum München, Munich, Germany; Institute for Medical Information Processing, Biometry, and Epidemiology (IBE), LMU Munich, Munich, Germany; Department of Sport and Health Sciences, Technical University of Munich, Munich, Germany; Department of Statistical Sciences, University of Bologna, Bologna, Italy; Centre for Health Economics and Policy Innovation (CHEPI), Imperial College Business School, London, UK; Department of Statistical Sciences, University of Bologna, Bologna, Italy; Institute of Health Economics and Health Care Management, Helmholtz Zentrum München, Munich, Germany; Department of Sport and Health Sciences, Technical University of Munich, Munich, Germany

## Abstract

**Background:**

The promotion of healthy lifestyles has high priority on the global public health agenda. Evidence on the real-world (cost-)effectiveness of policies addressing nutrition and physical activity is needed. To estimate short-term policy impacts, quasi-experimental methods using observational data are useful, while simulation models can estimate long-term impacts. We review the methods, challenges and potential synergies of both approaches for the evaluation of nutrition and physical activity policies.

**Methods:**

We performed an integrative review applying purposive literature sampling techniques to synthesize original articles, systematic reviews and lessons learned from public international workshops conducted within the European Union Policy Evaluation Network.

**Results:**

We highlight data requirements for policy evaluations, discuss the distinct assumptions of instrumental variable, difference-in-difference, and regression discontinuity designs and describe the necessary robustness and falsification analyses to test them. Further, we summarize the specific assumptions of comparative risk assessment and Markov state-transition simulation models, including their extension to microsimulation. We describe the advantages and limitations of these modelling approaches and discuss future directions, such as the adequate consideration of heterogeneous policy responses. Finally, we highlight how quasi-experimental and simulation modelling methods can be integrated into an evidence cycle for policy evaluation.

**Conclusions:**

Assumptions of quasi-experimental and simulation modelling methods in policy evaluations should be credible, rigorously tested and transparently communicated. Both approaches can be applied synergistically within a coherent framework to compare policy implementation scenarios and improve the estimation of nutrition and physical activity policy impacts, including their distribution across population sub-groups.

## Introduction

The promotion of healthy lifestyles has gained high priority on the public policy agenda over the last two decades. There is a growing demand for the credible estimation of policy impacts and evidence on the real-world effectiveness and cost-effectiveness of different population-based strategies addressing nutrition and physical activity.^1^^,^^2^ Yet, relative to clinical interventions, public policies are hard to randomize and it is thus a challenge to control for confounding factors and behavioural biases.^3^

Hence, quasi-experimental methods (QEM) using observational data for policy evaluation have become increasingly popular (table 1).^4^ Despite the availability of this quantitative toolbox, which is successfully applied in the social sciences, especially labour economics (see the 2021 Nobel prize in Economics, Royal Swedish Academy of Sciences, 2021), its application to identify causal effects of nutrition and physical activity policies on health outcomes is complex and potentially not fully exploited.^5^

**Table 1:** Google Scholar search of evaluation methods for nutrition and physical policies over three decades

Keywords	*N*, 1991–2000	%	*N*, 2001–10	%	*N*, 2011–20	%	Ratio 2011–20 vs. 1991–2000
Nutrition policy (total)	*5560* ^a^	*100*	*13 300*	*100*	*17 200*	*100*	*3.1*
Nutrition policy & randomized controlled trial	124	*2.2*	840	*6.3*	2820	*16.4*	*22.7*
Nutrition policy & quasi-experimental	50	*0.9*	253	*1.9*	812	*4.7*	*16.2*
Nutrition policy & difference-in-difference	1	*0.0*	41	*0.3*	186	*1.1*	*186.0*
Nutrition policy & simulation	299	*5.4*	553	*4.2*	1190	*6.9*	*4.0*
Nutrition policy & microsimulation	4	*0.1*	30	*0.2*	121	*0.7*	*30.3*
Physical activity policy (total)	*37*	*100*	*706*	*100.0*	*2640*	*100.0*	*71.4*
Physical activity policy & randomized controlled trial	4	*10.8*	74	*10.5*	565	*21.4*	*141.3*
Physical activity policy & quasi-experimental	2	*5.4*	75	*10.6*	287	*10.9*	*143.5*
Physical activity policy & difference-in-difference	0	*0.0*	0	*0.0*	25	*0.9*	*NA*
Physical activity policy & simulation	3	*8.1*	32	*4.5*	106	*4.0*	*35.3*
Physical activity policy & microsimulation	0	*0.0*	0	*0.0*	8	*0.3*	*NA*

^a^ Italic values indicate total amount of identified articles with the respective keyword.

Because the policy-behaviour-health causal link is probabilistic, delayed over time and the required data, particularly in the case of many confounding factors, may not be available, QEM cannot provide evidence on the long-term impact on health outcomes.^6^ Consequently, mathematical disease simulation models (SMs) projecting the long-term health and economic consequences are increasingly considered by scholars and policy makers (table 1).^4^^,^^7^^,^^8^

This article reviews QEM and SM approaches for the evaluation of nutrition and physical activity policies, their strengths and limitations, as well as their underlying general methodological assumptions. We show the complementarities of QEM and SM and discuss how their different characteristics could be exploited in a synergetic fashion to develop a more comprehensive concept of policy evaluation. We aim to provide guidance for applied researchers, policymakers and other stakeholders focussing on QEM and SM as two rapidly evolving methodological frameworks.

## Methods

We conducted an integrative review of assumptions, data requirements, strengths, limitations and synergies in the application of QEM and SM to evaluate population-based nutrition and physical activity policies. An integrative review approach enables the synthesis of diverse methods and types of information to provide a more comprehensive understanding of a research area. Integrative reviews are targeted and selective in nature and apply purposive literature sampling techniques.^9^^,^^10^ Thus, the aim, in contrast to a systematic review, is not to provide an exhaustive, systematic overview of a specific topic.

The starting point for our purposive searching comprised key original articles and systematic reviews identified by the author team within the European Union Policy Evaluation Network (PEN) project, which described the methodological assumptions and application of QEM and SM to evaluate population-based nutrition and physical activity policies.^8^^,^^11^^,^^12^ From these, we conducted purposive snowball searches to identify further key references based on subject matter expertise of the author team. The result of this approach does not represent a comprehensive list of all relevant original articles and systematic reviews, but a diverse selection of studies useful for exploring the strengths, limitations and applications of QEM and SM.

We defined QEM as all methods using observational data to estimate treatment effects (TEs) in the Neyman–Rubin counterfactual framework and SM as methods and techniques, which use mathematics to create abstractions of real-world phenomena with computer software from various sources of information.^13^^,^^14^

For each identified original article and systematic review, we extracted data on the general method (i.e. QM or SM), the specific type of method used or reviewed [e.g. difference-in-difference (DiD) analysis, Markov cohort SM], the underlying method-specific assumptions and limitations discussed and contextual information.

Additionally, we drew relevant data from the presentations of renowned scholars in QEM and SM at two public international workshops conducted within the European Union Policy Evaluation Network (PEN) project in Munich and Rimini in 2021 (materials available at: https://osf.io/fnmgk/ and https://osf.io/azf3n/).^11^

From these data sources, we synthesized key contemporary considerations in the application of QEM and SM. Specifically, we integrate an overview of QEM and SM methodology and summarize strengths and limitations, as well as the most important assumptions, future directions and synergies of both approaches in the evaluation of nutrition and physical activity policies.

## Results

### Quasi-experimental methods

Estimating the impact of a policy requires isolating the cause-effect path from a variety of confounding factors, i.e. causal inference.^12^ Outside the experimental setting, policy evaluation relies on observational data from so-called ‘natural experiments’ (NEs). Due to the lack of randomization, selection bias needs to be addressed to estimate the true policy effect.

We consider NE to be any setting where the statistical selection process, which determines whether subjects are exposed to the policy or not, is neither controlled, nor known by the evaluator and depends on uncontrollable external factors.^15^ The presence of uncontrollable external factors guarantees that the policy exposure is probabilistic. Although these probabilities are unknown and unknowable, this condition opens the way to statistical techniques for causal inference.^15^

This definition includes evaluations of nutrition and physical activity policies where exposure explicitly depends on subject characteristics, or because of indirect influences on participation.^15^ These factors might be measurable and available (e.g. residence, age and income), but also difficult to measure or not available (e.g. biological markers and psychological traits).

With NEs, exposure to the policy cannot be assumed to be independent from the outcome, as the external factors influencing the probability to be treated may also influence the outcomes. This means that the post-policy difference in the outcomes is a combination of policy impact and pre-existing selection bias.^12^ QEM control for this selection bias by design, so that after conditioning on the factors driving the assignment mechanism, the probability of being treated is independent from the potential outcomes, as in randomized controlled experiments (RCEs).

Impact estimation is relatively straightforward if all these conditioning variables are observed, an assumption, which is called selection on observables or unconfoundedness.^16^ However, this is hardly ever fulfilled. Beyond observables, data on relevant variables may be missing, or not accurately measured (e.g. psychological traits). These variables are called unobservables, and unbiased estimation of the policy impact implies the ability to control for both observables and unobservables.

### Testing assumptions and considering heterogeneous response

The fundamental QEM, instrumental variable models (IV), DiD and regression discontinuity designs (RDDs) control for both observables and unobservables, under certain assumptions.^17^ An extensive description of the methods is beyond the scope of this review and is provided elsewhere.^12^^,^^17^^,^^18^

We do not consider propensity score matching methods, which depend on the strongest formulation of unconfoundedness, as they require all relevant variables to be observable and any unobservable to be either non-relevant, or highly correlated with an observed variable. Hence, selection bias could be simply also addressed by a regression equation with the treatment status and all relevant covariates as explanatory variables.

Although implementing QEM methods is relatively straightforward with the appropriate (longitudinal) data, the real challenge lies in demonstrating that their underlying assumptions hold. Table 2 shows these assumptions for IV, DiD and RDD. Yet, in most cases no conclusive test exists and rigorous evaluations must present robustness and falsification analyses and support the credibility of their quantitative findings.^12^ Robustness analyses should demonstrate that relaxing one or more assumptions or changing analytical choices does not lead to substantial differences in the estimated policy impacts. Falsification analyses refer to the application of the methods to outcomes, target groups or time periods not affected by the policy, and should return non-significant estimates.

**Table 2:** Testing assumptions and dealing with unobservables in QEM

Method	Data requirements	Assumption allowing to deal with unobservables	Tests (examples)	Key references
Instrumental variables	Cross-sectional post-policy and at least one valid instrument	Relevance (of the instrument in determining the probability to be treated)	Testing probit model coefficient (Wald test significance not enough, *F*-statistic on the instrument coefficients should be large)	Cunningham (2021)^17^; Imbens & Rubin (2015)^12^; Davies et al. (2013)^19^
		Exclusion restriction: the instrument is exogenous	Lack of correlation between an excluded instrument and IV estimates of the residuals (non-conclusive and only feasible under overidentification)	
		Monotonicity: changes in instrument act in the same direction for all subjects	Not testable, and usually not important, but sensitivity analyses are possible	
Difference-in-difference	Repeated cross-sections: at least one cross-section before and one after the policy. Panel: at least one observation before and one after. Multiple observations before the policy needed to test the common trend assumption	Common (linear) trend vs. differential linear trend in the outcomes without the policy	Using data before-policy only, regress outcome on observables, a linear trend, and an interaction between the linear trend and the group variable (Wald test on the latter coefficient)	Cunningham (2021)^17^; Imbens & Rubin (2015)^12^; Callaway & Sant’Anna (2021)^20^
		Common (non-linear) vs. differential non-linear trends in the outcomes without the policy	Panel regression of outcomes on observables and fixed time effects, plus the interaction between the fixed time effects and the group variable, using before-policy data only. If there is a common trend, the interaction terms are all non-significant	
Regression discontinuity design (RDD)	Cross-sectional post-policy and an assignment-to-treatment variable related to the outcome. Data before the policy useful for sensitivity analysis.	Continuity assumption (no jump of the outcome at the cut-off without the policy—for fuzzy RDD also continuity of the probability of treatment)	Ideal check: run the same RDD on data before the policy and find no change at the cut-off. Alternative: RDD using the observables as the outcome, expecting non-significant results (non-conclusive)	Cunningham (2021)^17^; Imbens & Rubin (2015)^12^; Lee & Lemieux (2010)^21^
		Linearity assumption vs. non-linear functional forms	Not testable, but sensitivity checks are essential. Especially relevant for external validity. Ideal: test linear, non-linear (polynomial) and non-parametric specifications on data before the policy. Alternative: check robustness of the treatment effect estimate using different non-linear and non-parametric specifications, and different bandwidths.	

RDD, regression discontinuity design.

Under the appropriate conditions, not only can QEM be as effective as RCEs in eliciting the causal effect of policies, but they are potentially even superior in terms of external validity since they are free from some potential experimental biases (e.g. Hawthorne effect, sampling errors and compliance).^22^

A short but rigorous review of the key features and testing strategies for the application of QEM to public health studies is provided in Bärnighausen et al. (2017).^23^ These method-specific tests on assumptions are especially important from our perspective: (i) relevance and exogeneity in IV studies;^19^ (ii) test for differential non-linear trends in DiD studies, and their consideration (at least in robustness checks) if data allows;^20^ and (iii) the continuity assumption in RDDs, and the sensitivity of estimates to different functional forms and bandwidth selections.^21^

When estimating real-world policy impacts, it is important to consider that the actual impact—or TE—of the policy may be heterogeneous across exposed subjects, and average estimates (ATE) may thus be unsatisfactory. If subjects are exposed to the policy, but do not comply with the intervention, ATE estimates become problematic, as non-compliers are likely to systematically differ from both compliers and control subjects (i.e. reasons for compliance are correlated with TE). Consequently, two different TEs can be estimated: (i) considering all those exposed regardless of their compliance, which returns the average intention-to-treat effect; and (ii) considering treated subjects only, while accounting for the additional selection bias, which returns the local average treatment effect (LATE). When non-compliance is an issue, the LATE can be obtained through an IV estimator.^16^ Furthermore, TEs may be heterogeneous between subjects due to the nature of the intervention (e.g. personalized nutrition or physical activity programmes) since its effectiveness primarily depends on subject characteristics. Recently, there is a growing interest in methods (mostly based on machine learning) that capture this heterogeneity of policy impact across sub-populations, by letting the TE depend on sample covariates.^24^

### Applications and future directions

There are many examples of QEM successfully applied to the evaluation of nutrition policies.^25^ Applications to physical activity policies are less frequent but increased over the last few years (e.g. Xie et al., 2021 or Nakamura et al., 2021).^26–28^ The available methods are evolving together with the rising availability of large and detailed datasets on food consumption and physical activity. Specifically, consumer panels for food purchases and the emergence of innovative technologies for data collection over time (e.g. accelerometers and smartphone apps to measure physical activity) are valuable resources for QEM relying on longitudinal data. For example, synthetic control methods are a powerful approach when pre-policy data cover multiple periods and multiple non-treated groups (e.g. regions or states),^29^^,^^30^ while quantile DiD models and LASSO estimators may be of use for the estimation of heterogeneous treatment effects.^31^^,^^32^

### Simulation modelling

In the context of public health, SMs are usually used to simulate population health trajectories and the impact of health-related policies on risk factor trends, disease epidemiology, health-related quality of life and subsequent socio-economic consequences in populations using epidemiological and economic principles, but can also be extended to include macroeconomic and environmental aspects.^7^^,^^33–36^

For health policies that address unhealthy diets and physical inactivity as risk factors for non-communicable diseases (NCDs), such as type 2 diabetes, cardiovascular disease and cancer, these methods are of particular merit.^34^^,^^37^^,^^38^ Since these diseases are characterized by a chronic, progressing aetiology and their risk accumulates over time, effects of preventive policies are only measurable after many years, whereas the upfront political and policy implementation costs occur immediately.^39^

Beyond projecting epidemiologic health outcomes, SMs can estimate the long-term healthcare cost savings and non-health sector implications (e.g. lost productivity and environmental impact) of policies and are often applied within health-economic modelling to compare multiple policy scenarios, generating valuable information for priority setting.^4^^,^^40^ Finally SMs can provide policy impact corridors by simultaneously incorporating uncertainties from multiple sources.^41^^,^^42^

### Simulation modelling methods and main applications

Over the last decades, a variety of SMs in public health were applied in landmark projects, such as the Australian Assessing Cost-Effectiveness (ACE) in Prevention study, the US Childhood Obesity Intervention Cost-Effectiveness Study (CHOICES) project, the US Food Policy Review and Intervention Cost-Effectiveness (Food-PRICE) project (https://food-price.org/) and the Organization for Economic Co-operation and Development’s (OECD) Chronic Disease Prevention (CPD) modelling initiative.^38^^,^^43^^,^^44^

In this review, we cover the main SM approaches—from rather simple to highly complex—that are applied in the evaluation of nutrition and physical activity policies (table 3). An extensive discussion of SM for public health policy evaluation is available in Briggs et al. (2006), Briggs et al. (2016) and Emmert-Fees et al. (2021).

**Table 3:** Advantages, challenges and limitations of simulation modelling methods

Simulation modelling method	Data requirements	Advantages	Challenges and limitations	Seminal examples
Comparative risk assessment (CRA)	Population size and sex-age distribution; aggregated, stratified socio-demographic and epidemiological information on risk factors and diseases; risk factor–disease relationships; policy and intervention effectiveness	Easy to implement and low run times Straightforward communication to stakeholders Efficient integration of multiple risk factors and diseases	No explicit time component Only aggregate information Assumption of homogenous population No interaction and time-dependencies possible	Briggs et al. (2017)^45^; Collins et al. (2014)^46^
Markov (cohort) state-transition model	Population size and sex-age distribution; aggregated, stratified socio-demographic and epidemiological information on risk factors and diseases (incl. prevalence, incidence, case fatality and mortality); extensive data on risk factor–disease relationships to calculate transition probabilities; policy and intervention effectiveness	Comparably easy to implement with low number of health states Explicit time component (discrete steps) Allows for recurrence and looping Straightforward communication to stakeholders using figures Efficient integration of multiple risk factors and diseases (in combination with proportional multi-state life tables)	Only aggregate information Assumption of homogenous population Markovian assumption—no information on health status in previous time steps (no memory) Interaction and time-dependencies only possible for full (sub-)cohort and with complex model structures Complexity increases exponentially with number of health states	Cobiac et al. (2017)^47^; Vos et al. (2010)^43^; Carter et al. (2009)^48^
Microsimulation	Individual-level (repeated) cross-sectional or cohort data on socio-demographics and health behaviours from population health surveys; aggregated, stratified epidemiological information on diseases (incl. prevalence, incidence, case fatality and mortality); extensive stratified data on risk factor–disease relationships; policy and intervention effectiveness	Individuals instead of cohorts Explicit time component (discrete steps) High flexibility in model structure Allows for individual heterogeneity, complex interactions and time-dependencies Flexible estimation of various outcomes Can be used within CRA or Markov model framework	Can very quickly get very complex Communication with stakeholders can be difficult due to complexity Very high data requirements Very high computational requirements (especially with probabilistic sensitivity analyses) Limited by underlying model structure (e.g. CRA or Markov)	Kypridemos et al. (2017)^6^; Huang et al. (2019)^49^

CRA, comparative risk assessment. Information in table synthesized from Briggs et al. (2006), Briggs et al. (2016) and Emmert-Fees et al. (2021).

Comparative risk assessments (CRAs) are usually relatively simple cohort models, stratified by socio-economic and demographic groups, without explicitly accounting for time (table 3).^46^^,^^45^ First, risk factor and disease distributions are projected over the simulation period. In a second step, the effect of different policy scenarios on these projections is specified using population impact fractions to simulate outcomes.^7^^,^^50^

Markov state-transition models, particularly in combination with proportional multi-state life tables, are widely applied (table 3).^8^^,^^48^^,^^51^^,^^52^ Compared to CRAs, they explicitly model a population, often stratified in different age-sex-specific cohorts, over time. Markov models further implement explicit health states (e.g. healthy, sick and dead) between which cohorts transition proportionally, governed by epidemiological parameters, such as incidence, prevalence and case fatality rate.^41^^,^^47^

Microsimulation methods have become more common in recent years and are not a model type but rather a powerful technique that can be used within different modelling frameworks and embodies stochastic and dynamic components (table 3).^14^^,^^53^ In microsimulations, individuals with their own demographic, socio-economic and health profile are simulated over time instead of homogenous cohorts. Individual probabilistic health and disease trajectories are estimated based on risk estimates (the stochastic component) and updated sequentially over discrete time steps (e.g. years) while retaining all individual-level information (the dynamic component).

Beyond the types of SM discussed, there are other approaches and techniques each addressing specific analytical and contextual considerations, such as agent-based models, system dynamics models and discrete-event simulations, which are not yet widely used for the evaluation of nutrition and physical activity policies, though.^7^^,^^54–56^

### Conceptualization of models and required input data

Irrespective of the SM approach, four key interdependent components are needed to simulate the impact of nutrition and physical activity policies: (i) the level of complexity chosen to model risk factor–disease relationships; (ii) information on the (causal) relationship between risk factors, health and economic outcomes; (iii) demographic, socio-economic and epidemiological data; and (iv) the proposed mechanisms of policies.^8^

Most SM evaluations of nutrition policies rely on proximal risk factors, such as body mass index (BMI) and blood pressure, to estimate long-term NCD outcomes.^8^^,^^57^ While this is often a necessary simplification due to data requirements, evidence suggests that dietary quality, food processing and the food-specific combination of micronutrients may be equally important in the aetiology of disease. Currently, much of this complexity is not reflected in SMs.^58^ Correspondingly, it is essential to acknowledge differential effects of volume and intensity of physical activity when evaluating respective policies.^59^

Depending on the complexity of the model, the most important input for the simulation is the quantification of all explicitly included pathways between risk factors and outcomes. One challenge is that these are often only available as associations (i.e. non-causal) from non-randomized observational studies, potentially subject to unobserved confounding. This issue has been particularly discussed in nutritional epidemiology.^60^^,^^61^

Another central component of SMs is context/population-dependent demographic, socio-economic and epidemiological data. This includes prevalence and incidence data for diseases included in the model, as well as individual-level data on dietary intake and physical activity, particularly for microsimulations.^8^^,^^57^ Yet, many countries lack high-quality disease surveillance systems and national surveys needed to parameterize very complex models.

Understanding the actual mechanism of the policy under consideration including relevant externalities is crucial to integrate policy effects into SMs. This includes information (e.g. from QEM) on heterogeneous policy effects across sub-populations (e.g. sex, age, ethnicity and income), leading to differentiated simulation parameters, compensatory behaviour in response to the respective policy [e.g. change in snack consumption after introduction of sugar-sweetened beverage (SSB) tax], spatial aspects of policies (e.g. household and out-of-home consumption) and distributional effects to assess impacts on health inequalities.^62^

### Challenges and future directions

Two features are key to the implementation of SMs: (i) ‘validation’ and (ii) ‘transparency’.

The results of SM applications can only be as good as the model structure and input parameters. Model ‘validation’ is therefore essential for high-quality simulation-based impact evaluations. Validity dimensions include ‘input data validity’ (e.g. relative risks for disease, policy effects etc.), the ‘validity of the computational implementation’ of the model (e.g. code review) and its ability to predict data that was not used in building the model, such as national survey and surveillance data on risk factors and disease outcomes (‘external predictive validity’).^63–66^ However, simulated policy impacts are more difficult to validate as usually no observed data for comparison exists.

Due to the complexity of SMs, their assumptions and amount of data sources, it is crucial to ‘transparently’ provide information on results and methods for critical assessment. It is recommended to clearly communicate assumptions and publish lay summaries, detailed technical descriptions and computer code in Supplementary materials or online repositories.^65^^,^^67^ Addressing ‘validity’ and practicing ‘transparency’ is crucial to assure trust by policymakers.

General challenges, which should be considered include: (i) simulations over many years into the future are subject to secular trends, socio-cultural disruptions and unforeseen behavioural changes;^38^ (ii) differential dietary behaviours along socio-economic gradients are important to analyze equity impacts;^8^^,^^68^ and (iii) dietary behaviour is shaped by factors beyond health and systems thinking ideas could be incorporated into SMs to help determine non-health sector impacts of dietary policies (e.g. economy, education etc.).^54^

Future efforts to improve simulation modelling of nutrition and physical activity policies should aim to disentangle the direct, indirect and total effects of diet on health including environmental, behavioural and socio-cultural dimensions to more accurately estimate long-term policy impacts. Further, the influence of regional variation in food environments and consideration of out-of-home food intake may be another avenue for improvement.

Particularly, synergistic environmental impacts of nutrition and physical activity policies are of high relevance and may further increase stakeholder relevance across non-health sectors following a health-in-all-policies approach.^69^^,^^70^

## Discussion

QEM and SM can exploit valuable complementarities to inform policy makers on the impact and implications of different policy scenarios.^4^ Whereas QEM provide a robust way to evaluate the effect on selected (and mostly intermediate) outcomes of policy measures implemented in the past, SM provide a framework to generate projections of the wider and longer-term implications of policy scenarios, potentially including combination of policies that have not been jointly implemented before.^4^

We propose that evidence from QEM, RCEs, non-experimental epidemiological studies and SM should be understood as part of an evidence cycle for policy evaluation in which each method has its specialty and estimates from QEM can be used as inputs for the mathematical relationships in the SM, which help to identify, compare and prioritize outcomes, policy scenarios and impact dimensions. These might then in turn be subject to evaluation in QEM studies after a policy decision was made.

**Figure 1:**
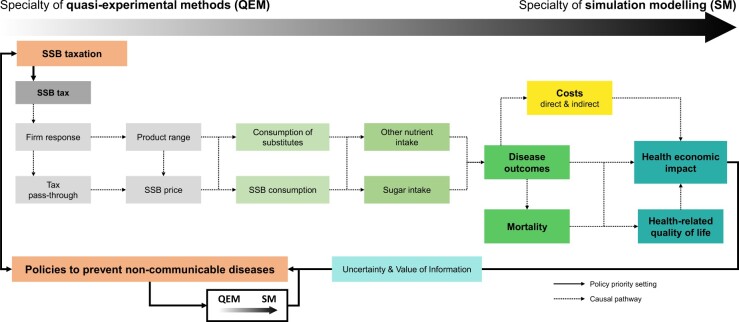
Exemplary policy evaluation and evidence cycle in the evaluation of a hypothetical tax on SSBs. SM, simulation modelling; SSB, sugar-sweetened beverage; QEM, quasi-experimental methods. Logic model of policy evaluation evidence cycle synergistically combining quasi-experimental studies and simulation modelling to inform policymaking

Figure 1 visualizes the nature of this evaluation cycle for an exemplary tax on SSBs, as introduced in several jurisdictions around the world.^71^

In this context, QEM can provide evidence of the tax impact on: (i) firm response (product range and possible reformulations, tax pass-through and price);^72–76^ and (ii) consumer response (including substitution patterns to other beverages and foods, at-home and out-of-home)^77^ and, potentially sugar and other nutrient intakes.^78^^,^^79^ One further key input needed in SM that QEM can provide are heterogeneous policy responses across firms and population sub-groups (e.g. CATE estimates).^80^

However, the estimation of intermediate and long-term health effects induced by changes in sugar and nutrient intakes through QEM is unfeasible, due to the lack of adequate longitudinal health data and the requirement for timely evaluations in policy making.^4^

SM approaches provide a solution to this challenge. They build on available survey data and the results of observational and QEM studies and, in the evaluation of an SSB tax, can translate changes in sugar intake and energy intake via established energy balance equations into changes in e.g. BMI.^81^ Using the causal link between BMI and other risk factors SM calculate population health trajectories of relevant NCDs, such as type 2 diabetes, cardiovascular disease and cancer.^49^ Ultimately, SM can project the expected long-term health and economic consequences of the SSB tax under consideration and compare alternative policies and taxation scenarios.^4^^,^^7^^,^^39^

Recent advances in causal inference for epidemiology emphasize the importance of integrating the plurality of methods for policy evaluation and have the potential to further strengthen the importance of QEM for public health simulation modelling.^82^^,^^83^ Furthermore, when the SM framework is grounded in systems thinking and formalized within a logic model, it can provide qualitative guidance on the priorities for additional QEM studies for those parameters with insufficient evidence.^4^ In the future, highly complex simulations, may model pathways from the consumer to health and non-health sectors to evaluate policies. Here, the method of value of information analysis—a technique that assesses the expected gain from reducing uncertainty in key parameters—could even be used to prioritize the estimation of model input parameters within a formal economic framework.^41^

## Conclusion

QEM and SM have distinct strengths and limitations as standalone frameworks to estimate the impact of nutrition and physical activity policies. This integrative review analyzed a selective list of critical elements and assumptions to be considered when implementing these methodologies, and proposes to synergistically combine QEM and SM to overcome their limitations.

Below, we summarize the main lessons drawn:

- Assumptions behind models must be transparent and credible. This implies rigorous testing whenever possible, and validation through recognized robustness checks and sensitivity analyses.
- Nutrition and physical activity policies may act rapidly on behaviours, but the health effects may only become apparent in the longer term. QEM are a powerful tool to identify immediate causal effects, SMs are a better suited to project these behavioural changes into long-term outcomes.
- The growing interest in targeted policies and the variability in individual response, call for the application of QEM and SM to allow for heterogeneous responses, and consider the distribution of impacts across different population sub-groups.
- The implementation of multi-component lifestyle policies is a major challenge for QEM to elicit the contribution of individual measures. However, their joint application with SM has the potential to generate new evidence on the effectiveness of multi-component policies.

Finally, the evolution in methods for policy impact evaluation is closely related to the availability of adequate data. Until recently, the application of QEM and SM to nutrition and physical activity policies has been hindered by limitations in the quality and quantity of (longitudinal) data. Novel data technologies can help generate new evidence, and extend the toolkit for policy evaluation.

## Data Availability

No new data were generated or analyzed in support of this research. Key points Quasi-experimental methods are useful to identify short-term causal effects of nutrition and physical activity policies. Simulation models can be used to project the long-term health and economic impacts of nutrition and physical activity policies. Quasi-experimental and simulation modelling methods have strengths and intrinsic limitations as standalone frameworks. Combining the strengths of both approaches synergistically can lead to a more comprehensive approach to public health policy evaluation.
